# Report on the Progress of Pathology, Practical Medicine, and Therapeutics, during the Years 1842-3-4

**Published:** 1845-07

**Authors:** 


### 4. Diseases of the Vascular System.

*Heart.* The following are the principal conclusions to which M. Aran has been led by his "researches on general pericardial adhesions."†† When entire and free from any trace of recent inflammation, the patient may be free from all suffering. Derangement of the respiratory and circulatory functions depends more on the alteration of nutrition which the heart undergoes than on the adhesions themselves. Dilatation with hypertrophy is one of the most

‖ London Med. Gazette, p. 696, vol. i, 1843-4.

** Provincial Medical and Surgical Journal, Jan. 6, 1844.

†† Archives Gén. de Méd. 1844, p. 466.

immediate consequences of general, non-cellular, pericardial adhesion. He has never met with an instance to the contrary. The pathognomonic sign of adhesions *is a diminution or extinction of the second sound.* Three cases are given in which the diagnosis thus founded was confirmed after death. The second sound is diminished not only in clearness, but also in duration and extent, and this in proportion to the intimacy of the adhesions and the amplitude of the cavities of the heart. In old cases, it is extinct over the whole præcordial region, and even over the whole chest, so that the first sound (a little prolonged) is immediately followed by the interval of rest, also somewhat prolonged. In insufficiency of the aortic valves, the second sound is heard to the right of the heart; and at the posterior part of the chest, and over the præcordial region, it is replaced by a prolonged and sometimes musical blowing sound ("bruit de soufflet aspiratif"). He explains the diminution or extinction of the second sound by referring to the impediments existing to the heart's free action, and the consequent imperfect dilatation and contraction of the ventricle, in which circumstances neither the usual shock of the return of the column of blood on the aortic valves, nor the return of the fibres of the ventricle to their passive state, can occur with sufficient force to produce the second sound. Professor Forget* is unable to lay down any constant or invariable sign of general adhesion, and has never met with the epigastric depression during the systole, described by M. Sandras. The presumed relation between adhesions and hypertrophy he thinks rational, but requires confirmation, and that it is not proved that adhesions alone, when old, may not produce dropsy and death, by long-continued impediment to the heart's action.

*Hypertrophy of the left ventricle in old people.* M. Dubrueil† thinks that in many cases dilatation of the aorta is the consequence of the loss of elasticity of the vessels which attends old age, and that this loss of elasticity explains why hypertrophy should be the almost constant attendant on aortic aneurism. When the movement communicated to the blood by the arteries is enfeebled in consequence of their morbid condition, the heart compensates for it by increased action, which after a time induces hypertrophy. Hence, in most old persons, we find that hypertrophy of the left ventricle coincides with ossification (?) of the vessels, when this occupies a certain extent of the arterial system.

M. Fauvel,‡ in a memoir entitled 'On the Stethoscopic Signs of *narrowing of the left Auriculo-ventricular Opening*,' after noticing the different existing opinions, states that he had observed the abnormal sound, attending the first sound of the heart, in certain cases, to precede the impulse. In consequence of the occurrence of some cases, the details of which are given, his attention was more closely directed to this subject, and he has been led to the conclusion that a "bruit de râpe," situated at the apex of the heart, to the left, and immediately preceding the first normal sound, may be the only morbid sound corresponding to very considerable narrowing of the left auriculo-ventricular orifice, without valvular insufficiency. This presystolic abnormal sound corresponds with the contraction of the auricle at the moment the blood is driven into the ventricle, across the diminished orifice, and therefore situated as it is, at the apex of the heart, to the left, it is the *most probable* stethoscopic indication of narrowing of the mitral valves.§ Dr. Hamernjk|| attempts to show that inflammation of the substance of the heart is much more frequent than has been thought, since the alteration of tissue consequent on fibrinous exudation is only to be detected by the microscope; by the naked eye it may be confounded with fatty degeneration. When the fibres connected with the mitral or tricuspid valves are thus altered by inflammatory exudation into their substance, they cannot contract with sufficient force, and thus the phenomena

* Gazette Médicale, April 1844.

† Observations sur les Anevrismes, &c., par J. Dubrueil; 8vo, Montpelier, 1842. Gazette Méd. Sept. 17, 1842.

‡ Archives Générales de Méd. Jan. 1843.

§ M. Aran coincides with these views. Vide Arch. Gén. de Méd. Nov. 1842.

|| Oesterreich. Méd. Jahrbüch. July and Aug. 1843.

of *valvular insufficiency* may be induced. He gives a case in which the musculi papillares of the mitral valve were found atrophied, flattened, and to a great extent converted into cellulo fibrous tissue, from infiltration of lymph. Mr. Moore O'Ferrall remarks that uncomplicated obstruction of the aperture is not necessarily attended by a murmur; that this may disappear after a time, and that the diagnosis can be made only by the observation that a well-marked systolic murmur had previously existed in combination with the general symptoms of the disease.*

*Hydro-pneumo-pericardium.* An example of this form of disease is given by M. Bricheteau,† in which fluctuation of liquid in the pericardium, coinciding with each beat of the heart, was perceptible by the ear during life, and resembled the flap of a paddle against the water. The pericardium was found after death greatly distended, and being punctured, gave exit, with audible sound, to a quantity of fetid gas, and a considerable quantity of very fetid sero-purulent fluid. (Bricheteau has referred to authorities, and finds his case almost unique, the only *complete* case of the kind.)

*Digitalis, in certain diseases of the heart.* Professor William Henderson‡ confirms the observations of Dr. Corrigan (Ed. Med. and Surg. Journal,) that in a permanently patent state of the aortic valves, the prolonged use of digitalis is injurious, inasmuch as frequent contractions of the heart are, in this state, necessary to overcome the tendency to regurgitation, the cause of the hypertrophy. On the other hand, in diseases of the left auriculo-ventricular valves, increased frequency of the heart's pulsations increases the dyspnea, and the symptoms consequent on obstructed circulation, partly in consequence of the hypertrophy of the right ventricle, and partly owing to what takes place at the diseased orifice. If this be merely narrowed, more frequent closing of the valves will increase the impediments to the onward course of the blood, and if in a patent state, will augment the regurgitation—in either case increasing the dyspnea. Hence digitalis, by diminishing the frequency of the heart's pulsation in this form of disease is beneficial in diminishing the dyspnea and augmenting the size and force of the pulse, which from being small and feeble, will become full and firm, when the medicine has reduced the heart's contractions to 40 or 50 per minute. M. Debreyne§ has derived the best effects from the following plan of treatment in organic affections of the heart, excepting in cases where the pulse is very slow or feeble, while the extremities are cold, asphyxia imminent, the countenance livid, and the swelling considerable. "After local or general bleeding, or leeches to the anus, according to circumstances," he prescribes tincture of digitalis in gradually augmented doses, and with each dose, dissolved in the same vehicle with it, ʒj of nitrate of potash. The medicines produce no good effect unless given in doses sufficient to cause nausea and vertigo. The sedative power of nitrate of potash on the heart he considers proved by Aran's researches.

### 5. Diseases of the Nervous System.

*Cerebral Auscultation.* Dr. Whitney,|| of Newton, Massachusetts, states that in ausculting the heads of healthy children, four distinct sounds are heard, produced respectively by the acts of respiration and deglutition, and by the voice and action of the heart; these are sometimes so altered in character in diseases of the encephalon, as to become symptoms of cerebral disease. A cephalic bellows-sound, or modification of the natural sound of the heart, is heard in various diseases. It has been noticed in cerebral congestion and inflammation, hydrocephalus, compression of the brain, ossification of the arteries, and the hydrencephaloid disease. Encephalic, or cerebral œgophony, has been noticed only in cases attended with effusion and extravasation in and about

* Dublin Journal of Medical Sciences, July, 1843, p. 418.

† Archives Gén. de Méd., March, 1844, p. 334; par M. Bricheteau, Médecin de l'Hôpital Necker.

‡ Northern Journal of Medicine, May 1844.

§ Bull. Gén. de Thérapeut. 15 et 30 Dec. 1842.

|| American Journal of Med. Sciences, Oct. 1843.

the substance of the brain. A purring thrill has been heard in aneurism of the basilar artery, and a cooing or musical sound is considered pathognomonic of anæmia of the brain.

*Cerebro-spinal meningitis, epidemic.** During the last few years a terrible disease has prevailed in different towns in France, attacking principally the common soldiers of the garrisons of these towns, namely Versailles, Lyons, Avignon, Bayonne, Givet, Metz, Strasbourg, Nancy, and more recently in other localities. M. Faure Villar† has described the disease as it prevailed at Versailles. M. Gassaud‡ has given a very similar account of its characters as it appeared at Bordeaux. M. Gasté§ has published his experience of the disease at Metz, in 1840; MM. Forget and Tourdes‖ have observed it at Strasbourg; and M. Chauffard** at Avignon. Lastly, M. Rollet†† describes its appearance at Nancy. The symptoms resemble very closely those of inflammation of the membranes of the brain and spinal cord in sporadic cases. According to M. Rollet, the disease occurs in two forms; in the one which he denominates "méningite encéphalo-rachidienne," there are no signs of lesion of the nervous centres themselves; no affection of sensation or motion; though there are all the symptoms of inflammation of the membranes; at first, rigors, then malaise, tinnitus aurium, vertigo, violent pain in the head, extending along the vertebral column; agitation or restlessness, and slight delirium and moderate fever, or absence of fever. In the second form there is affection of the intellectual faculties, and also of the functions of motion and sensation, and more or less complete abolition of all the senses. This form of the disease is illustrated by the following case: "When the patient was admitted into the hospital, the face was dusky, (cyanosé,) the eyes fixed; the sclerotics injected; the pupils dilated and insensible to the action of light; there were furious delirium; wild cries; constant movements of the limbs; trismus; retraction of the head backwards, and marked diminution of sensibility; the skin was rather warmer than natural; the pulse 80, full, and hard; the tongue dry, and red at the tip; and the patient scarcely able to protrude it from the mouth. The next day there was profound stupor, nearly complete deafness; unintelligible muttering elicited by questions, and complete loss of sensibility. Death took place on the third day. The patient had been bled twice; forty-six leeches had been applied, and an *oxyerst* to the forehead. *Autopsy.* The cerebral arachnoid was very vascular; and upon the whole surface of the pia mater and the brain there was a layer of purulent plastic matter; and a considerable collection of this matter at the base of the brain, about the pons varolii and medulla oblongata. The cerebrum was slightly punctated but not softened. The choroid plexus injected: the cerebellum softened; and the arbor vitæ of a blood-red colour. Beneath the spinal arachnoid there was the same kind of purulent matter as was observed beneath the cerebral arachnoid, and opposite the third dorsal vertebra, a considerable collection of pus, and also about eight grammes opposite the last dorsal vertebra. The substance of the spinal cord appeared healthy." The lesions here described are exactly the same as those mentioned by MM. Faure-Villar, Chauffard, and Forget. Morbid changes from inflammation have also been observed in the alimentary canal, but M. Rollet regards them as mere accidental coincidents, while M. Forget attaches great importance to them. Again, M. Villar noticed that lumbricoid worms were very frequently found in the intestinal canal in fatal cases, in 42

* Rapport sur une Mémoire de Cérébro-rachidienne et de l'encephalo-méningite épidémique, par M. Rollet, Médecin en chef de l'Hôpital Militaire de Nancy. (M. Ferrus, rapporteur.) Bulletin de l'Acad. Roy. de Méd. Oct. 15, 1842, t. viii, p. 43.

† Mémoires de Médecine et de Chirurgie Militaires, 1840, t. xlviii. ‡ Ibid.

§ In a tract entitled Mélanges de Médecine.

‖ Relation de l'Epidémie de Méningite Encéphalo-rachidenne observée à Strasbourg, par M. Forget. Paris, 1842. Hist de l'Epidémie de Méningite Cérébro-spinale, observée à Strasbourg en 1840-41, by M. Gabriel Tourdes, Paris, 8vo, 1842.

** Mémoire sur les cérébro-spinites qui ont regné in 1840 et 1841. Revue Médicale, Mai 1842.

†† Sixième Observation de M. Rollet.

out of 56; and M. Gassaud cites cases in which they passed from the patient during life. But M. Rollet observed this complication only twice at Nancy. He remarks, that in those cases in which the substance of the brain is affected, there is an almost continual tendency to intermission, or at least remission, which alternates about every three hours with an exacerbation, but he regards this merely as characteristic of the encephalo-meningitis, not as an evidence of the disease being of the nature of remittent fever, which is the view taken by M. Gassaud.

With regard to *treatment*, M. Faure-Villar tried all rational methods, but declares that none seemed superior to the rest. Out of 154 cases which he treated, 66 terminated fatally. M. Gassaud, who regarded the disease as a "fièvre céphalalgique subintrante," produced by marsh miasm, affirms that, of 162 soldiers attacked only two died when he had begun to treat them with medium doses of sulphate of quinine, at the same time that he employed purges, and at the commencement, venesection. M. Forget recommends the antiphlogistic plan of treatment at the commencement of the disease, and afterwards opium. Of 40 cases, however, he lost 24. M. Chauffard failed to cure the malady by antiphlogistic means, the most prompt, direct, and energetic; by revulsives, purgatives, calomel, and also by various tonics. Opium triumphed over it. But it was necessary to give it in large doses. The medicine most advantageously combined with opium was the sulphate of quinine. Before this plan was adopted, only 1 case was cured out of 30; afterwards the disease was less fatal than it ordinarily is in sporadic cases. Lastly, M. Rollet found that all the cases of simple cerebro-spinal meningitis, (that is to say, of inflammation of the membranes without lesion of the nervous centres themselves,) yielded to simple but energetic antiphlogistic treatment, or at most to this treatment aided by counter-irritants to the skin. One remedy only could control the more violent cases of encéphalo-meningitis, when the brain and spinal cord also suffered, and this was cauterization. (In one case which is detailed, the actual cautery was applied at twelve distinct spots along the spine, beside counter-irritants.) M. Tourdes states that, of 195 soldiers attacked, 122 died. He agrees with M. Chauffard that bleeding, tartar emetic, mercurials, refrigerants, and revulsives, were of no avail; but he cannot confirm all that Chauffard has said in favour of opium.

Dr. Gillkrest,* in a Report to Sir James M'Grigor, has described a similar epidemic which prevailed among the civil population of Gibraltar from January to May 1, 1844. Of 16,000 inhabitants 450 were attacked, of whom 190 had symptoms of more or less gravity, and 42 died. The majority of the cases occurred between two and fifteen years of age, and few only were attacked in a severe form above the age of thirty. With but few exceptions the disease prevailed among the indigent classes. "There is no question" says Dr. G. "of the identity of the symptoms in our cases, with those described in the Versailles epidemic." No special atmospheric peculiarities could be assigned as its cause. Previous to its breaking out, an epidemical catarrh which had prevailed, declined; and before its setting in, as well as during its prevalence, "it was remarked that in indispositions of any kind, there was a tendency to headache more or less severe, the occiput being oftener than usual the seat of pain, and the muscles of the back of the neck being affected with aching." No opportunity was afforded of examining the head of a single child; in the adult cases the dura mater was always found free, but the arachnoid and pia mater exhibited the most unequivocal marks of inflammation, especially at the base of the brain, where pus as well as lymph, was frequently found. The ventricles in some instances contained large quantities of serum, lymph, and pus. Mercury and the ordinary antiphlogistic means constituted the treatment.

Several cases of *sporadic spinal meningitis* presenting many points of in-

* Medical Gazette, July 5, 1844.

terest, have been recorded. One by Dr. Eitner, fatal in four days.* Professor Wagner† has detailed a remarkable case in which universal suppuration of the cerebro-spinal membranes existed without any corresponding symptoms, which were those of gastric derangement, till two days preceding death, when convulsions occurred. There was general softening of the brain and spinal cord.‡ Dr. Drazic has reported a case occurring under the care of Prof. Skoda, in which there was general paralysis of the voluntary muscles, without any loss of sensation or affection of the sensorium. The day before death there was profuse sweating, paralysis of the diaphragm, dyspnea, with general mucous rhonchus (but no paralysis of the bladder), and subsequently trismus and convulsive movements of the muscles of the face. The brain was found to be natural. Between the membranes of the cord was a little grayish clear serum; the whole spinal cord was somewhat atrophied as well as the roots of the motor nerves, the upper ones especially being soft. The substance of the cord was generally pale and firm.§

*Apoplexy, contraction of the limbs in.* M. Durand Fardel‖ has investigated that condition of the muscles occurring in hemiplegia, in which though deprived of voluntary motion, contraction to a greater or less extent, either temporary or more permanent occurs. From his researches he concludes, that in cerebral hemorrhage contraction of the paralysed or non-paralysed limbs, almost invariably accompanies the rupture of the apoplectic cavity into the ventricles or between the membranes; that contraction rarely attends hemorrhage limited to the substance of the hemispheres; and, lastly, that contraction is a very frequent symptom in cerebral hemorrhage.

The object of Dr. Mayo's Lumleian Lectures** is to illustrate the views of Dr. Kirkland respecting "those cases of apoplexy in which vascular plethora, congestion, or extravasation, has not occasioned the attack." "They are all marked by suddenness of invasion, but some are more vehement, others milder"—"suddenness of invasion, and the absence of evidence of prior arterial excitement, or effusion, are the diagnostics on which Dr. Kirkland principally relies, as identifying his nervous apoplexy. It may coexist with, or be promptly followed by sanguineous or serous effusion; but these are not its causes." To illustrate the character of this form of apoplexy Dr. Mayo relates 5 causes (one original,) and remarks on the absence of any evidence of antecedent vascular fulness or effusion, and that the two cases which terminated most favorably were those in which the treatment had been most sedative. He then proceeds to illustrate Dr. Kirkland's views in relation to the paralytic affections which are "not brought on by compression, suppuration, or any mechanical cause," and which are described as "spontaneous or true palsy from sudden loss of nervous power." Dr. Mayo, however, considers that in the treatment of the 'apoplexia nervosa gravior' Dr. Kirkland has pushed his principles too far, and that this form may tolerate and even require more depletion than even cases of presumed extravasation. He suggests the hypothesis that the vital power is not, as Dr. Kirkland supposed, destroyed, "but under a temporary interruption." Meanwhile the heart continuing to perform its functions, this supposition would imply a subsequent danger of congestion a tergo and a liability to rupture; so that though no arterial action or sanguineous extravasation should precede or accompany it, the case may require the same treatment in kind, as such phenomena would have indicated. M. Gay (in 1808) attempted to show that no such disease as sanguineous apoplexy exists, that bleeding is injurious in all cases of apoplexy, but that emetics are indicated *in every case.* His essay has been translated by

* Med. Zeitung, 21 Dec. 1843. † Oesterreich. Med. Wochens. Nov. 4, 1842.

‡ See also Arch. Gén. de Méd. Fev. 1843. Observation d'un Cas remarkable d'affection de la Moelle Epinière, etc., par M. Gérard, de Marseilles. § Oesterreich. Méd. Wochens. Jan. 21, 1843.

‖ Archives Gén. de Médecine, 1843, p. 300. De la Contraction dans l'Hemorrhagie Cérébrale, par Dr. Max. Durand Fardel. ** Med. Gazette, Nov. 11 and 25, and Dec. 2, 1842.

Mr. Copeman, who has appended some observations on the subject of bleeding in apoplexy.* Dr. T. Reinboldt† thinks that few cases of apoplexy are benefited by large bleeding, but that small doses of from ℥ij to ℥iv are useful in assisting the brain to recover its powers.‡

*Hemiplegia from obstructed circulation.* Three cases of great interest, have been recorded, in which hemiplegia was induced by sudden obstruction to the arterial circulation within the cranium; in two instances occasioned by ligature of the carotid artery, and in the third by the consequences of dissecting aneurism of the aorta. M. Sedillot§ tied the common carotid to arrest hemorrhage from a wound behind the right branch of the lower jaw. Complete hemiplegia of the right side of the face and of the left side of the body followed the operation, and the patient was deprived of intelligence so far as scarcely to be able to comprehend questions put to him. He died nine days after the operation, and the right side of the brain was found somewhat diminished in consistency, and deprived of its due proportion of arterial blood. In Dr. Fairfax's case, hemiplegia of the opposite arm and leg followed a similar operation, but there was no paralysis of the face.|| The case related by Dr. Todd** presents many points of interest. A stout plethoric man, æt. 37, was suddenly seized with syncope and afterwards complained of violent pain in the loins, along the course of the ureters, thighs, and abdomen, attended with some tympanitic swelling, nausea, and scanty urine. Despite of treatment the kidneys ceased to act; hemiplegia of the left side succeeded; feebleness of pulse of the right side; bellows-murmur in the course of the aorta and innominata; feebleness of respiration in the right lung; drowsiness and sluggishness. About the fifth or sixth day the secretion of urine returned, the pupils which had been unequal became equal, and some power of the paralysed side was regained. Signs of internal hemorrhage however came on, and the man died suddenly on the eleventh day from the seizure. A copious effusion of blood was found in the pericardium derived from a small aneurismal sac, communicating with the aorta. From this spot the blood had formed a new channel for itself, splitting up the middle coat along the aorta and innominata, and the right carotid for some distance, and then plugging up the latter vessel, and arresting the circulation through it. The right hemisphere of the brain was found exsanguineous, and all that part above the fissure of Sylvius (supplied with blood by the middle cerebral artery) exhibited numerous patches of softening, without discoloration, implicating the white as well as the gray matter. [A case was recorded some time ago, by Mr. Vincent, in which the patient died on the seventh day after ligature of the right carotid, with hemiplegia of the left side. Pale softening of the right hemisphere was discovered after death. Such cases, independently of their important practical bearing, are deserving the attention of those who would refer softening of the brain in all cases, either to inflammation or post-mortem changes.]

*Hemiplegia connected with syphilis.* In the 'Medical Gazette' for May 27, 1842, Dr. Budd called attention to some cases of paralysis concurrent with, or depending on the presence of the syphilitic virus in the system. Mr. Inman†† has since met with five similar cases. A fatal case has been published by Dr. Todd, where the post-mortem examination revealed inflammation of one cerebral hemisphere, and red softening of the other. In sup-

* Essay on the Nature and Treatment of Apoplexy, by M. Gay. Translated by E. Copeman, esq. with an Appendix; London, 1843.

† Ueber d. Schlagfluss von Dr. Th. Reinbold, Hanover; in Schmidt's Jahrbücher, No. 3, 1844.

‡ As a counterpart to certain recent modes of treating phthisis by paracentesis, the following almost equally rational proposition may amuse the reader: M. Claudius Barbier, of Lyons, after adducing (as though he believed it original) the hypothesis of the unalterable fulness of the cerebral vessels, to show the futility of bleeding in apoplexy, gravely proposes the application of the trephine before having recourse to venesection!! He does not, however, appear as yet to have put his notable project into execution. Journal des Conn. Méd. July 1843.

§ Gazette Médicale, Sept. 3, 1842.

|| Medical Gazette, p. 351. Vol. i, 1843-4.

** Trans. of the Med. and Chir. Society.

†† London Medical Gazette, July 21, 1843.

port of the view that these are not mere coincidences, Mr. Inman remarks that in all his cases, as well as in the four related by Dr. Budd, and in Dr. Todd's case, the patients were under forty years of age, whilst ordinarily apoplexy attacks only persons who have passed that age. [The author of this Report met with a case, some years ago, where an apoplectic attack, followed by hemiplegia, occurred in a young man æt. 24, who was at the time suffering from secondary symptoms for which he had been taking mercury. As the hemiplegia of one side gradually disappeared, the opposite side became affected, but ultimately the patient recovered.]

*Paraplegia.* Sir B. Brodie* states that in chronic affections of the spinal cord, producing paraplegia, &c., the treatment must, to a certain extent, be empirical; but that he has never seen any beneficial results from the use of counter-irritation. On the contrary, he has often seen it productive of mischief. It is essential that the bowels should be kept open, and that the black tary secretions which take place, should be got rid of. For this purpose he recommends pills consisting of compound extr. of colocynth, with croton oil. The treatment he has found most successful consists of the exhibition of pills containing gr. j. zinci sulphatis, thrice a day, with a draught containing ♏ xx tinct. cantharadis. These should be persevered in for some time, gradually increasing the dose, but not to any great extent. In other cases, he has seen small doses of bichloride of mercury useful, which does not, he thinks, act as mercury, but much in the same way as sulphate of zinc. Mr. Gorham† and Dr. Badeley‡ have each reported a case of paraplegia, in which the recovery was attributed to strychnia.

*Paralysis treated by electro-galvanism.* Several cases of paralysis have been related where the cure was attributed to electro-galvanism; one of complete paralysis of the œsophagus of long standing, followed by hemiplegia; the particulars of which are detailed by Sir A. Knight,§ a second, of hemiplegia of three years' standing, described by Dr. Martin Lynch;|| a third, of complete paraplegia succeeding to ptosis and amaurosis, which had been treated by mercury to a considerable extent, recorded by Mr. Howell, of Clapton. In this case, however, the cure was not complete, and the electro-galvanism was conjoined with quinine, iron, and tonics.**

*Hydrocephalus in youth.* Dr. Henry Kennedy†† concludes a paper "On Hydrocephalus which occurs at a particular period of life," by throwing into a set of propositions the different points to which his attention has been drawn. That an affection of the brain of the hydrocephalic character is not at all unfrequently met with between the ages of twelve and twenty-five years. That it is more common in females than males, in the proportion of two to one. That in the majority of cases it commences with symptoms of mild fever. That it sometimes begins by a distinct complaint of the head, for some days, the patient being still able to go about. That when the disease commences by fever, the first signs of anything going wrong take place very commonly at night; and a marked increase of fever may then be observed. That during the progress of the disease the pulse exhibits the characters of hydrocephalus, and to a marked degree. That alterations about the eye (strabismus, &c.,) are often among the earliest symptoms, pointing out that mischief is coming. That the pathology of the affection is confined in great part to the arachnoid at the base of the brain, with more or less effusion into the ventricles. That there are grounds for supposing the inflammation to be of a specific character, probably strumous. That when the affection has fully declared itself, the treatment has yet to be determined; but local bleedings, with mercury and blisters, hold out the best prospect of success. In connexion with this subject

* Lancet, vol. i, 1843-4, p. 427. † Ib. May 11, 1844. ‡ Medical Gazette, July 12, 1844.
§ Dublin Med. Press, March 29, 1843; from Provincial Medical and Surgical Journal.
|| Provincial Med. and Surg. Journal, March 23, 1844. ** Med. Gazette, vol. i, 1843-4, p. 481.
†† Dublin Journal of Med. Science, July 1843, p. 382.

some observations have been given by Dr. J. B. S. Jackson,* entitled 'Tubercular Meningitis in the Adult.'

*Epilepsy.* M. Leuret† having always under his care, 100 or more male epileptics, has given the results of his researches on the causes, symptoms, course, and termination of epilepsy. Among predisposing causes, his tables show that adolescence must be ranked, and as young children are often carried off by early attacks, and few admitted into hospital, he considers childhood also a predisposing cause. Hereditary predisposition could be traced in 7 only of 106 cases. In reference to the real or presumed causes, of the 106, 39 could not assign even a probable cause, but of the remainder 35 assigned fear; 12 onanism; 6 drunkenness; 2 anger; and 2 falls. M. Leuret thinks the influence of the depressing passion of fear cannot be questioned. Seven cases are detailed in illustration, and great stress laid on the danger of exposing children to the influence of fear, as of all causes of epilepsy this he thinks the best established. In 82 of the 106, the attacks occurred at regular periods, and then were rarely single, but recurred frequently for twelve or twenty-four hours. The latter cases are often quickly fatal. In reference to the influence of season, the tables indicate that cold is injurious, and heat favorable. The relation of the moon's changes to 70 cases watched through the whole year, show that this luminary exerts no influence whatever on the course of epilepsy. The electric state of the atmosphere is not without influence; the attacks being more frequent in stormy weather. Intemperance and onanism are frequent causes of the return of an attack. Those in whom the seizures have observed regular periods, suffer in various ways if at the regular time they have not an attack.

A case is related by Dr. Parrish,‡ in which an epileptic paroxysm immediately succeeded to a blow on the head. After a lapse of eight months the disease returned, the attacks became frequent, and were immediately preceded by severe shooting pains over the seat of the original injury, though no pain was complained of at other times. This spot was tender, and firm pressure excited severe pain and general nervous agitation. An incision was made over the tender portion of the scalp, an issue established and kept up for seven weeks, when the soreness had entirely disappeared. From this time the patient had no return of the disease. Dr. William Heise,§ of the Connaught Lunatic Asylum, states, that an epileptic lunatic who had received a severe wound of the head, with fracture of the skull, was from that time cured of his epilepsy and restored to reason.

*In epilepsy and epileptical mania,* Dr. Sharkey|| thinks there is a particular tolerance of digitalis, which acts as a pure sedative; the characteristic effects on the circulation being manifested in the course of twenty-four or forty-eight hours. He gives the tincture in doses of from ʒij to ℥ss, and has detailed two cases of mania associated with epilepsy, and one of maniacal excitement in which marked benefit followed large doses of this medicine. In another case of epilepsy, great tolerance of the remedy was manifested, and relief obtained, with sound sleep and subsidence of the excitement. [In a case inwhich, on the periodic return of epileptic attacks, the patient is scarcely free from the paroxysms for twenty-four hours, the Reporter has tried the effects of digitalis in doses of 40 min. every three or four hours, on three occasions, with, apparently, considerable benefit.] Dr. Baretti** has detailed four cases of epilepsy successfully treated by the valerianate of zinc. Dr. Debreque†† has found the extract of belladonna by far the most successful remedy when there are no symptoms of cerebral congestion. When the paroxysms occur at long intervals, the belladonna is administered for a week or two before the

* New England Quarterly Journal of Medicine and Surgery, Oct. 1842.

† Archives Gén. de Méd. 1843, p. 32. Recherches sur l'Epilepsie, par M. Leuret, de Bicêtre.

‡ Philadelphia Examiner, vol. vi, No. 2.

§ Dublin Medical Press, Sept. 6, 1843.

|| Medical Gazette, vol. i, 1843-4, p. 305, and vol. ii, p. 340.

** Bulletino delle Scienze Mediche, Feb. and March, 1844, p. 121.

†† Thérapeutique appliquée, &c. par P. J. C. Debreque, 2de Ed. 1844.

expected invasion, and if preceded by a distinct aura, a strong dose of ammonia is recommended, as serving to ward off the paroxysm. *

*Delirium tremens.* The following remarkable case of delirium tremens, is given by Mr. S. Flood, in which, after trying opium fully, with tartar emetic, digitalis, &c., without effect, belladonna was employed in the following way. A large blister having been applied between the scapulæ, the cuticle was stripped off, three inches long and two wide, and a plaster of pure extract of belladonna applied to the denuded surface. The man was, at the time, in a state of furious delirium, with contracted pupils, pulse 110, weak, and very irritable; and had not slept for 360 hours. So acute was the pain produced by the plaster, that he was instantly subdued; and entreated its immediate removal. In three minutes he ceased to complain; in five minutes there were slight twitchings of the muscles of the face and arms, his utterance became indistinct, he kept up a stupified laugh like a man much intoxicated; the pupils began rapidly to dilate, and in seven minutes were open to their fullest extent. He now became very drowsy and begged to lie down; the belladonna therefore, was sponged off, simple ointment applied, and he then fell back on his pillow, and in nine minutes from the first application was in a profound sleep, which lasted for seven hours. During the sleep, which was free from stertor, the pulse fluctuated remarkably. At the commencement it was 110, small and irritable; in five minutes it rose to 140; and in twenty minutes to 160; then gradually fell, till at the end of six hours, it had sunk to 108, and was full and soft. At the end of seven hours he awoke quite quiet, but after staring about him in stupified astonishment, soon relapsed into his former state of wildness. Opiates were now tried again in large doses, but without effect, and as he was apparently sinking from prolonged excitement, belladonna was applied, in the same way, a second time, two days after the first application. The same chain of phenomena followed, and sound sleep was induced, which continued for nine hours and a half. On the following day, belladonna was a third time applied, but to the same surface; from this time he gradually improved. Dr. Fosgate† recommends the union of ammonia with opium, not only as aiding to sustain the powers of the system, but also as modifying the influence of opium, diminishing its poisonous and increasing its therapeutic action.

*Affections of the brain and spinal cord, connected with acute cardiac diseases.* The dependence of certain affections of the brain and spinal cord on acute disease of the heart, now very generally admitted, has been farther illustrated in the Lumleian lectures of Dr. G. Burrows.‡ From various sources he has collected sixteen cases: 1, some having all the usual symptoms of inflammation of the brain and its membranes; 2, cases simulating mania and dementia; 3, others characterized by apoplectic and epileptic symptoms; 4, another class with well-marked symptoms of tetanus and trismus; and five others, presenting symptoms of aggravated hysteria and chorea. It has been supposed that such cases are only met with in connexion with rheumatism, and especially when pericarditis is ingrafted on articular rheumatism; but of the cases collected by Dr. Burrows no rheumatic affection could be discovered in six. In the twelve fatal cases no trace of disease could be detected, in either the brain or its membranes, a fact opposed to the supposition that metastasis is the cause of the symptoms. Dr. Burrows coincides with Dr. Bright's opinion, that the nervous symptoms are of the same character as those produced by irritation of the gums.

*Chorea.* A case of chorea, occurring in a girl æt. 16, is related by M. Trousseau,§ the fatal termination of which will not surprise British practitioners. The girl had had two previous attacks, which are said to have ceded to the use of strychnia. The disease, however, returned after a fright, and in an

* Lancet, vol. ii, 1842-3, p. 12. Vide also Ibid. p. 897, for further remarks on the action of Belladonna.
† American Journal of Medical Sciences, Jan. 1844.
‡ Medical Gazette, May 26, 1843.
§ Bulletin. Gén. de Thérap. t. xxv, p. 226.

aggravated form, the symptoms being very severe, and attended by violent convulsive movements of the trunk, and opisthotonos. Strychnia was again tried, and persevered with for four days, but failed to afford any relief. Morphia was then tried, but the symptoms became more and more aggravated and terrible, till the third day, when death occurred. The brain and spinal cord were carefully examined, but nothing whatever abnormal was detected. [Purgatives seem never to have been mentioned, or thought of.] M. Rougier.* however, details ten cases, in children, successfully treated by strychnia. In all, the disease was at first exasperated. In the majority, tetanic convulsions occurred, sometimes of a very alarming character, but always allayed by drinking a glass of cold water ! To be successful, M. Rougier thinks the remedy must, in the first instance, either produce tetanic symptoms, or sensibly augment the involuntary movements.

In anæmic cases, after exhibiting purgatives and iron† Dr. Hildreth gives full doses of sulphate of quinine. Cases presenting indications of cerebral determination and congestion, are considered unfit for this treatment. Dr. H. also strongly recommends the cimicifuga racemosa, in doses of ʒj—ʒij of the saturated tincture, or a strong decoction with spices and brandy. Four cases of a convulsive disease allied to chorea have been recorded in the American Journals, under the name of "*salaam convulsion.*" One occurred in the person of a boy æt. 6, and is detailed by Dr. Ezra P. Bennett.‡ In January 1842, the lad had a slight attack, marked by sudden convulsive action of the right leg and thigh, followed by temporary loss of motion. A month afterwards the disease returned in a severer form, presenting the following characters : " The leg and arm of the right side were in a state of tonic spasm, the left leg and arm in constant motion of flexion and extension, and his head in violent motion backwards and forwards, as far as it could possibly go." The spasms were violent, lasted from two to three minutes, were productive of severe pain, and left him paralysed. He was perfectly conscious. Purgatives, blisters, the warm bath, and cordials were of no benefit. Two grains of opium every two hours produced some alleviation and some sleep. Strychine in doses of $\frac{1}{20}$ gr. augmented the spasms to such a degree as to endanger the boy's life. Two large blisters along the spine, at first augmented, but subsequently relieved the spasms. The little girl, æt. 7, whose case Dr. Barlon§ records, was seized every few hours with spasmodic jerking of the extremities forwards, and a bowing of the head, with instantaneous relaxation, repeated at intervals of a few seconds, and accompanied with a quick expiration and noise, such as would be produced by a blow on the epigastrium. She was cured by purgatives. This peculiar nodding motion of the head has given the name of "salaam convulsion" to the disease.

*Catalepsy. Thirteen years' standing.* Professor Huss, of Stockholm,‖ states that a woman, æt. 30, at the age of 12 was suddenly deprived of consciousness, to which succeeded loss of speech and hemiplegia of the right side, lasting a year. She then recovered, and menstruated at 17; after which she became subject to attacks, during which " she became stiff as a poker," preserving the position in which the attacks found her. They began with palpitations and tinnitus aurium, the eyelids being spasmodically closed; but when opened, the pupils were dilated; the limbs were extended, and to the patient felt stiff, though easily bent by the observers, but retained the position in which they were placed; the neck was bent back, the face red, but the features unaltered; the carotids beat strongly. After some minutes she heaved several deep inspirations, and gradually recovered. Consciousness was retained during the attacks, but she could neither hear, nor speak, nor feel when touched. She recovered by treatment directed to restore the catamenial functions and the use of the

* Journal de Méd. de Lyons, July 1843.
† American Journal of Med. Sciences, Jan. 1843.
‡ American Journal of Med. Sciences, April 1843.
§ Ibid. Jan. 1843.
‖ Gazette Méd. de Paris, Feb. 4, 1844.

sesquic. ferri. The catalepsy was unassociated with any exstatic or other peculiar psychological phenomena, and was manifestly connected with the uterine derangement.

*Hydrophobia.* M. Dupuy* has related to the Acad. of Med. the history of a case, where a person bitten by a mad dog escaped hydrophobia by having the wound freely cauterized. At the same meeting it was stated that at Martinique, 18 individuals were bitten by mad dogs during one year; that 17 of these had their wounds freely cauterized, and did not afterwards suffer; but that the 18th, who did not submit to this operation, was seized with hydrophobia M. Lerou, of Dijon, has also announced, as the result of his experience, that in that town, all who had been bitten by mad dogs and had their wounds freely cauterized, have escaped, whilst all those who were not so treated have fallen victims to hydrophobia. M. Robert,† who distinguishes between hydrophobia and true rabies, thinks the latter (often accompanied by the former) is never cured. He has detailed minutely the post-mortem appearances of a fatal case, occurring to a girl æt. 12. These exhibit nothing unusual, except that the blood was extremely pale and not coagulated, and the inferior two thirds of the spinal cord much softened.

M. Allier, recollecting the power of compression of the carotids in cases of epilepsy, compressed both arteries in a case of hydrophobia, just as a paroxysm of convulsions was coming on, which immediately ceased, and the patient fell into a kind of syncope. Not being allowed to persevere with his plan, the patient died.‡

*Tetanus.* A case of idiopathic tetanus, occurring suddenly on the decline of an attack of simple inflammatory fever, is recorded by Dr. A. Robert, of Chaumont.§ The patient recovered after copious bleedings, purgatives, blisters, antispasmodics, and opiates. [It seems questionable whether idiopathic tetanus is a correct designation for such a case.] Dr. Purefoy‖ relates a case of trismus supervening on the extraction of a molar tooth, and cured by bleeding, tobacco enemata, and calomel and opium; and Dr. Hutchinson, of Nottingham, two cases of tetanus cured by belladonna, a trial of which he recommends in hydrophobia.**

*Tic douloureux.* Dr. Hunt's†† treatise contains much practical information on the varieties and treatment of tic douloureux and other neuralgic disorders, the result of considerable observation of those affections in the warm and humid climate of the south of Devon. He arranges the various forms of tic douloureux according to the causes from which it springs: 1, arising from the neuralgic habit; 2, from dyspepsia; 3, from dyspepsia complicated with congestion of the liver and other viscera; 4, from anæmia; 5, morbid action in the spine; 6, disorder of the uterus; 7, disease of the brain; 8, local mechanical causes; 9, malaria, recession of eruptions, and other causes. His treatment is directed, in the first instance, to the removal of that morbid condition which appears to stand in the relation of an exciting cause, and of course varies in each case. He proposes no new mode of treatment, but points out the indications, and gives excellent rules for the administration of well-known remedies, more especially of arsenic, in which he places great confidence as a tonic. He finds it of most efficacy in those of a lax fibre, languid circulation, cold and moist skin, and whose urine is pale and plentiful.

M. Banneix‡‡ lays stress on the existence of one or more spots, painful on pressure, along the course of the affected nerve, as important to the diagnosis

* Med. Gazette, Jan. 19, 1844; from Journal de Pharm. † Gazette des Hôpitaux, Nov. 1, 1842.

‡ Gazette des Hôpitaux, Nov. 1, 1842. [This plan of compressing the carotids to arrest convulsive actions was recommended and adopted by Dr. Parry in hysteria.]

§ Archives Gén. de Médecine, Sept. 1843, p. 92. ‖ Dublin Medical Press, Sept. 6, 1843.

** Lancet, May 25, 1844.

†† The Nature and Treatment of Tic Douloureux, &c., by H. Hunt, M.D.; London, 1844.

‡‡ Bulletin Gén. de Thérap. t. xxv, p. 17.

of neuralgia, and after reviewing the principal modes of treatment, concludes by recommending repeated blisters over the different painful spots, as ascertained by pressure, and as an adjuvant, the application of the salts of morphia to the denuded surface. He thinks the connexion between neuralgia and morbid states of the digestive organs has been overrated. Dr. Hutchinson recommends the internal and external use of belladonna;* and Dr. Dangerfield has related two cases confirmatory of Dr. H.'s views.†

Dr. Roelants of Rotterdam,‡ for six years past has obtained the most successful results from nux vomica in the treatment of prosopalgia; 29 cases are detailed, both recent and confirmed, of whom 25 were cured, 3 were under treatment, and 1 was but partially treated. The curative action is speedily manifested, and the duration of the affection appears to have no influence in this respect, very chronic cases being much relieved in eight days. In one case of seven years' standing, in a man æt. 61, two thirds of a grain of powdered nux vomica were given every two hours, followed by great relief in eight days, and a complete cure after two months' treatment. For five months longer (i. e. after the disappearance of all pain,) he continued to take three grains per diem to prevent a relapse, which after three years had not occurred. The importance of continuing the treatment for some time is particularly insisted on. This man had taken iron largely without effect. M. Rougier of Lyons§ employs morphia in the treatment of neuralgia, by the endermic method, and then exhibits strychnine internally to remove the partial paralysis that sometimes ensues, and to confirm the cure. If the strychnine reproduce or increase the pain, it is a sign that the neuralgia is not effectively cured, and vice versa.

*Sciatica.* Dr. Fioravante|| was led to employ blisters to the heels in the treatment of sciatica, from hearing of several empirical cures performed by a woman who applied irritating substances (ranunculus sceleratus,) to the same parts. The epidermis was softened, and then removed, till the blisters would produce their ordinary effects. The suppuration thus established was kept up for some time in chronic cases. Twelve cases are mentioned which were speedily cured by this means. [A memoir was published about ten years ago by Dr. Petrini of Aguila, a town of the Abruzzi, to make known a method of treating sciatica by the application of the actual cautery between the little toe and the next one of the affected limb. Quadri of Naples has obtained great success, by adopting the same treatment, and states that a Capuchin monk, affected with sciatica, used to carry about with him a cauterising iron.]

### 6. Diseases of the Urinary Organs and Skin.

Dr. Barlow** has collected a valuable series of cases illustrative of the diagnosis of disease of the kidney, in which he points out the value of irritability of the stomach as a distinguishing symptom between diseases of the kidneys, accompanied with irritation, and affections of other structures in the neighbourhood. The paper also contains much information elucidatory of the peculiar cerebral affections depending on the non-depuration of the blood by the kidney.

*Albuminuria, Causes of.* M. Fourcault's†† valuable investigations into the effects of suppression of the cutaneous secretion, have shown that albuminuria can be thus readily induced. He supposes that this effect is produced by the excess of lactic acid which is then found in the blood, and which reacts on the albumen. The introduction of lactate of soda into the veins also produces albuminuria, by favouring the excess of lactic acid in the blood. When the acid secretion of the skin is suddenly checked, it produces a marked change in the organic elements of the blood; and when gradually suppressed, a number

* Lancet, vol. i, 1843-4. † Ibid. Oct. 7, 1843.
‡ Arch. Gén. de Méd. Sept. 1843; from Alg. Konst. Letterbode. No. 10, 1843.
§ Gaz. Méd. de Paris, July 8, 1843; from Journal de Méd. de Lyons.
|| Annali Universali di Medicina, Nov. 1843. ** Guy's Hospital Reports, vol. vii.
†† Comptes Rendus, May 5, 1844.

of chronic diseases are produced, among which are albuminaria, scrofula, lepra, &c. &c. He admits, however, that albuminuria may also, though rarely, originate in a primary affection of the kidneys. Dr. Meyer of Tubingen,* concludes from his researches, that albuminuria may be produced by an accumulation of blood in the kidneys, (without any organic alteration of their structure) either from augmented arterial supply, or stagnation in the veins. In this way he accounts for albuminous urine in diseases of the heart and lungs; and his conclusions are deduced from five experiments on animals, in some interrupting the flow of venous blood, and in others tying the aorta below the origin of the renal arteries; in all of which cases the urine became albuminous.

— *Pathology and Treatment of.* Dr. G. O. Rees,† assuming that most observers are now agreed that the blood-corpuscle consists of a membranous sac inclosing colouring matter, has directed attention to the extreme tenuity of the blood in certain stages of the morbus Brightii; and shown how this condition constitutes the true cause of the deficient proportion of hematosine observed in the later periods, inasmuch as it must interrupt those endosmotic changes occurring between the contents of the corpuscle and the chyle, when each fluid possesses its ordinary specific gravity. The increased quantity of water circulating in the early stages, he considers to be caused by the discharge of albumen by the kidneys. The iron which colours the contents of the corpuscles he believes to be communicated by the *aqueous extractive* of the chyle which passes into the corpuscle by endosmosis; and this process being interrupted by the abnormal tenuity of the blood, the red corpuscles are diminished. In a subsequent paper,‡ after pointing out the analogy in the pathology of various forms of anemia, to morbus Brightii, he recommends in the early stages the same plan of treatment that is found beneficial in chlorosis and anemia from loss of blood, viz. chalybeate tonics, saline purgatives, and nutritious diet, which though not immediately calculated to remove the condition of kidney known to exist, he has found efficacious in preserving the normal state of the blood, and thus assisting in recovery. He condemns any attempt to relieve the nephritic congestion by depletion, but recommends counter-irritation and dry cupping. Numerous instances have been recorded of granular degeneration of the kidney, even in an advanced stage, unattended with albuminous urine; and of persistent albuminous urine independent of any structural disease of the kidney.§

An instructive series of cases illustrative of albuminuria, arranged so as to exhibit the influence of particular remedies or plans of treatment, has been published by Drs. Bright and Barlow.|| Dr. Alken** has found hydriodate of potash and iodine ointment useful; and Dr. Gutbrod†† having observed in the Vienna hospital great benefit from iodine, tried the ioduret of iron in two well-marked cases in the advanced stage, and with the best results. M. Monneret obtained great improvement in one case from tinct. cantharidis, in doses increased up to 60 drops; and in another case, from the use of vapour baths.‡‡

*Diseases of the Skin.* In his Treatise on Diseases of the Skin, Mr. E. Wilson§§ has adopted a natural system of classification, based on the anatomy and physiology of the skin, and given full information on all that is new and important connected with recent investigations in this department of medical

* Archiv für Physiolog. Heilk. Jan. 1844.

† Guy's Hospital Reports, April 1843.

‡ Medical Gazette, Aug. 16, 1844.

§ See the following among others: Edinburgh Med. and Surg. Journal, April 1844; Wells's Report of Malta Hospital; Dublin Journal of Med. Science, Jan. 1843, and clinical lectures by Dr. Graves; Oesterrcich. Med. Wochen. Nov. 26 and Dec. 3, 1842.

|| Guy's Hospital Reports, 2d series, No. i.

** Oesterreich. Med. Wochens. Jan. 28, 1843.

†† Gaz. des Hôpitaux, Sept. 7, 1843; from Correspond. Blatt des Wurtemburg. &c.

‡‡ Gazette des Hôpitaux, Oct. 13, 1842.

§§ A Practical and Theoretical Treatise, &c. by Erasmus Wilson, 1844, 8vo.

science. Scarlatina, rubeola, variola, varicella, and vaccinia, are classed together, under the head of inflammations of the dermis and mucous membrane, characterized by constitutional symptoms of a specific kind. [That there is a general resemblance between these diseases, in many important particulars, is admitted; but few, perhaps, will be disposed to agree with Mr. Wilson, that the contagious virus from which they originate and spread, is in all of them identical. Whatever view may be taken of the relation between variola and its kindred diseases, varicella and vaccinia, far more satisfactory proofs than any Mr. W. has given, are required to establish the identity of the virus generating smallpox, measles, and scarlet fever.]

Dr. Veiel,* superintendent of the Cronstadt hospital for diseases of the skin, has arranged these diseases into two classes: eruptions of the blood (blutflechten), and eruptions of the skin (hautflechten). The first are but symptomatic of internal derangements of the system, of some dyscrasis of the blood; the second are idiopathic diseases of some of the elementary tissues of the skin. These two classes, he maintains, are alike distinguishable by their causes, phenomena, consequences, and treatment. Excess of albumen gives rise to eczema; excess of fibrine to impetigo, and of the aqueous part of the blood to chronic urticaria, lichen, &c. The 'Nouvelle Dermatologie' of M. Beaume contains the exposition of a system founded on the etiology of cutaneous diseases.†

*Elephantiasis Græcorum* is endemic in Norway, and Dr. Danielsen states‡ it to be so prevalent that, of a population of 200,000, 1200 are lepers. He represents it as not contagious, but hereditary; especiallythe tubercular form, which has been seen in the newly-born infant. It is inevitably fatal. It occurs in two forms: el. tuberculosa, and el. anæsthetica. When not transmitted, the conditions favouring its evolution are damp and dirty clothes small, ill-ventilated houses, thick fogs, indifferent food, and the other accompaniments of poverty. Mr. Trompes§ has ascertained that, throughout the Sardinian dominions there are about 100 lepers, and that the disease is undoubtedly contagious after it has reached the stage of suppuration. M. Benet, formerly physician to the King of Lahore, attributes the disease chiefly to the eating of pork and salt fish. Like other writers, he considers the preparations of arsenic the best remedies, and especially recommends an Indian formula, viz. 105 grains of white arsenic triturated for four days with 6 times as much black pepper, of which pills of the size of the seeds of "tares," are given night and morning.|| Mr. Skene, of the 52d foot, has described the disease as seen in New Brunswick,** where the first case occurred in 1817. He ascribes it to a special virus of unknown origin, but spreading by hereditary transmission and contagion.†† Dr. Boyle, however, of St. John's, denies that there is any evidence of the disease having been transmitted by contagion.

M. Grüby has described certain cryptogamic plants, to the formation of which he attributes the origin and spread of *porrigo decalvans* and *porrigo scutulata*. In porrigo decalvans, the parasite appears in the form of chaplets of sporules (rarely branched,) which surround the hair as with a sheath, for several lines beyond its exit from the skin. The hair first becomes gray, then brittle, and so falls off in pieces. In porrigo scutulata the sporules fill the roots and shafts of the hair, become surrounded with articulated filaments that extend up the interior of the hair, which thus becomes filled, is rendered brittle, and breaks off. The sporules in this instance are evolved in the piliferous follicles, whilst in porrigo decalvans they are generated on the hair after it has left the surface of the skin.‡‡ Dr. Wigan recommends the application of Beaufoy's concentrated acetic acid

* Medicinisches Correspondenz Blatt, (in Gaz. Méd. de Paris, 15 April 1843.)

† Nouvelle Dermatologie, &c. par P. Beaume, Paris, 1842, 2 vols. 8vo.

‡ Comptes Rendus, April 1, 1844. § Annales de Thérapeutique, Nov. 1843.

|| Rapport fait par M. F. Le Gros, sur un travail de M. Benet; Gaz. des Hôpitaux, Dec. 24, 1842.

** Lond. Med. Gazette, June 14, 1844. †† Ibid. late date.

‡‡ Séance de l'Acad. des Sciences, Aug. 14, 1843, and Comptes Rendus, April 1, 1844.

for three or four minutes to the scalp, previously shorn, as an unfailing remedy, and also as a sure method for detecting the disease in parts apparently sound;* and Dr. Furnival verifies Dr. Wigan's statements.†

M. Emery‡ has given some practical observations on *sycosis menti* and its treatment, which he thinks should be strictly antiphlogistic. He confines himself to the application of emollient cataplasms and alkaline lotions, and condemns the use of stimulating ointments. M. Duchene Duparc advises that the pustules of sycosis and of acne should be painted night and morning with a concentrated solution of sulphuret of potash; and M. Dauvergne, after commencing with poultices, vapour douches, and antiphlogistic diet, &c., recommends the local application of a solution of sulphate of iron.§ In the course of some practical remarks on the use of arsenic in cutaneous diseases, Mr. Erichsen|| states that it should be given only to persons of a debilitated, relaxed habit, free from any symptoms of gastric irritation, when the disease is in an indolent, passive state, and especially when local stimuli produce no permanent irritation.

### 7. DISEASES OF UNCERTAIN SEAT, &C.

*Gout and Rheumatism.* Dr. Bence Jones's treatise,** in which he endeavours to apply the physiological and chemical doctrines of Liebig to the elucidation of the pathology and therapeutics of gravel, calculus, and gout, has already been noticed in this Journal. Dr. Todd,†† though admitting the humoral origin of gout and rheumatism, denies that lithic acid is the materies morbi in gout, which must, he thinks, be looked for as a compound derived from the unhealthy action of the stomach and duodenum, and which being taken into the blood, unites with elements of bile that have accumulated there, through defective secretory action of the liver. The copious deposits of lithic acid often observed in the urine for weeks or months without the occurrence of gout, he thinks sufficiently prove that lithic acid cannot be the materies morbi, and in like manner he infers, from the non-existence of lithic acid in excess, in the urine in certain cases of gout, "that the morbid element of the disease may be present independently of lithic acid;" and he particularly insists that low, depressed states of the system are favorable to the development of the gouty paroxysm. Rheumatism he believes to consist in the presence of the same morbid element (lactic acid) in the blood, and calls attention to the important fact that the rheumatic diathesis may exist without presenting the usual phenomena of rheumatism, and that in this condition the heart may become seriously affected. The cardiac inflammation may in fact be primary, and when co-existing with the articular affection, is not usually to be viewed as the result of metastasis. He devotes a chapter to the connexion between rheumatism and uterine derangement, and adduces important reasons for believing that the accumulation of rheumatic matter in the blood may be the result of defective uterine action.

M. Briquet having employed with advantage sulphate of quinine in the treatment of typhoid fever, has had recourse to it in acute rheumatism. In his memoir, read to the French Academy,‡‡ he has detailed 23 cases treated in the following heroic manner: On the first day, 4, 5, or 6 grammes (ʒj to ʒiss.) of the sulph. quinæ (according to the age, &c. of the patient) were given, suspended in mucilage, in divided doses in the course of twelve hours. The same doses were repeated on the second and third days, when the symptoms had usually abated, and the doses were gradually diminished by grs. xv. per diem. The average duration of the pain and swelling of the joints was from three to five days. In more than one third there was cardiac complication,

* Medical Gazette, Sept. 15, 1843.

† Idem, vol. i, 1843-4, p. 16.

‡ Bull. Gén. de Thérap. t. xxv, p. 170.

§ Idem, t. xxiv, 15 et 30 April, 1843.

|| Med. Gaz. May 5, 12, & 19, 1843.

** On Gravel, Calculus, &c. by H. Bence Jones, M.B.; Lond. 1842.

†† Croonian Lectures, by R. B. Todd, M.D. 8vo, 1843.

‡‡ Séance, Oct. 15, 1842; Gazette des Hôpitaux, Nov. 17.

recent or chronic. In all but four there was a marked abatement of the symptoms in twenty-four hours. The date of the affection did not influence the cure. Relapses occurred in two only. M. Devergie, in testing Briquet's statements,* began with smaller doses, and gradually increased them, and made trial of the same remedy in chronic cases. He confirms Briquet's views, except that in acute cases he would give smaller doses than in the chronic. Other examples of the efficacy of Briquet's plan may be found scattered through the French journals, and Signor Mascheroni treated 40 cases in the Lodi hospital† with the best results, two or three only presenting any cardiac affection. The general result, however, of the investigations to which Briquet's memoir has led, is decidedly opposed to both the safety and utility of his plan. Several fatal cases have occurred in the French hospitals,‡ from these heroic doses. The conflicting opinions in reference to the toxical effects of large doses of quinine induced M. Melier to investigate the whole subject afresh, and Messrs. Andral, Beguin, &c. have reported on the memoir presented by Melier to the French Academy.§ His experiments sufficiently prove the poisonous effects on dogs, of large doses, viz. gr. 15 and upwards. The blood was always found fluid, and the brain, lungs, and gastroenteric mucous membrane congested. The symptoms in men and dogs are similar, viz. intoxication, disturbance of the senses, diarrhœa, hæmaturia, amaurosis, deafness, (very frequent,) aphonia, delirium, coma, epileptiform, convulsions, and death. [These statements correspond with those of Giaccomiri, as the result of his experiments; 'Annali Univers. di Medicina,' March, 1841.] Melier shows that the utility of moderate doses of quinine in certain forms of rheumatism had been long ago pointed out by other physicians, e. g. Morton, Leroy, &c., and the reporters refer to Haygarth's clinical researches, who obtained the best results from doses of gr. 10 and upwards of *bark* every four hours. Dr. Popham's observations on this subject‖ induce him to believe that bark is most useful in the fibrous form of rheumatism, and after the more acute symptoms have been combated by antiphlogistic means. If cardiac symptoms are present, the bark should be deferred till these are overcome. Periodicity of the symptoms, whether produced by the treatment or peculiar to the attack, calls for bark, and especially when profuse colliquative acid sweats are present, and the pulse small and feeble. Dr. J. J. Furnival** contends that acute rheumatism consists essentially in an acid state of the blood, and that the best treatment consists of the use of alkalies and antiphlogistics, since adopting which, he has never met with a single example of cardiac complication. The treatment of rheumatism by large doses of nitre has also attracted much attention. M. Martin Solon†† appears to have been led to this mode of treatment by the observations of Brocklesby, Macbride, and others, and by the consideration of the contra-stimulant, temperant qualities of the salt. Since 1840 he has thus treated 33 cases of severe acute rheumatism, demanding active means, of which 20 were cured from the second to the seventh day of treatment. Nitre, he states, is easily tolerated by rheumatic patients in doses of from ʒv to ʒxv, in the 24 hours, if given in large quantities of diluent drinks. It is in acute cases only that it is useful, and its sole apparent effects are diminution of the heat of the skin and of the frequency of the pulse. It prevents the occurrence of endocarditis, and shortens the period of convalescence; but in complicated cases does not supersede the necessity for bloodletting. M. Monneret,‡‡ however, in an instructive memoir on the comparative effects of treatment by colchicum, nitre, and bloodletting, states that the influence of nitre on the progress of eight severe cases appeared absolutely null. Neither the heat of

* Gazette Médicale, Dec. 30, 1842. † Gazetta Medica di Milano, Feb. 1843.

‡ L'Examinateur Médicale, t. iii, No. 16; and Gaz. des Hôp. 11 April, 1843.

§ Bulletin de l'Acad. Roy. de Méd. 31 May and 15 June, 1843.

‖ Dublin Medical Journal, Sept. 1844. ** Lancet, June 1, 1844.

†† Bull. de l'Acad. Roy. &c. t. ix, p. 130. See also, for further observations on the nitre treatment, Allgem. Med. Cent. Zeitung, 25 Mar. 1843, par Dr. C. F. Bartels.

‡‡ Arch. Gén. de Méd. March 1844, p. 269.

skin nor quickness of pulse was in the least affected. Professor Forget,* on the contrary, contends that nitre in large doses is a remedy of real efficacy *in certain cases,* and that in doses of from 8 to 45 drachms, given with diluents, it is rarely productive of any ill consequences. M. Requin's experiments† are strongly corroborative of the efficacy of Dr. Corrigan's treatment by opium, but do not justify the abandonment of depletion.

*Diabetes mellitus.* Dr. Percy's essays‡ contain the results of some inquiries, very ably conducted, into the changes which starch and wheaten flour undergo in the healthy stomach—the situations in which grape-sugar is found in diabetes—what part of the system it is formed, and what are the particular conditions attending its formation. He believes the kidneys merely eliminate the sugar from the system, that the disease may exist independently of any structural change, and that sugar is certainly formed in the stomach in diabetes. Dr. Watts§ considers the proximate cause of the disease to reside in the stomach, which in the first stage is in a state of inflammation, during which lactic acid is secreted, and lactic acid and lithate of ammonia are present in the urine; in the latter stage the stomach is in a state of atony, and sugar and lactic acid abound in the blood and all the secretions. Four or five bodies of diabetic patients, examined by Dr. Watson,|| presented no evidence of the usual connexion with scrofula.

Several examples of reputed cures of diabetes are recorded. One by Mr. Hodges of Downpatrick,** in which the exciting causes appear to have been loss of blood, and a diet consisting almost exclusively of potatoes and other vegetables. The treatment consisted of the use of ammonia and nitrogenised food, according to Dr. Barlow's plan. A second by Dr. Gennaro Festeggiano,†† in which the treatment consisted of drinks containing a small quantity of ipecacuanha, and acidulated with hydrochloric acid. The symptoms abated in eight days, and the patient was cured in a month. A third, by M. Combette,‡‡ in which the rapidity of the cure was equally remarkable. The patient, a man æt. 40, under Mr. Rostan's care, had been treated for a month, without benefit, by an exclusively animal diet and vinous lemonade. M. Combette put him again on animal diet, with a very little bread, and prescribed a pill, containing 25 centigrammes of iodide of iron four times a day. Four days after, the urine was very considerably diminished, and from that time he continued rapidly to improve. A fourth case is related by Dr. Cowan,§§ in which animal food, with the cruciferous vegetables and muriated tinct. of iron were the principal remedies. This case remarkably illustrated the injurious influence of bread as an article of diet, which also was remarked in an unsuccessful case detailed by Dr. Theoph. Thompson.|||| A fifth case is reported by Dr. Grayson to have been cured by the tinctures of cinchona, valerian, and lytta, animal food, with lime water and a little wine for drink.*** MM. Mialhe have detailed a case of 18 months' standing cured by bicarbonate of soda and hydrated calcined magnesia, flannel clothing, and vapour-baths.

*Purpura febrilis, fatal in 28 hours.* M. Hummel††† has related a case with this title, occurring to an athletic man who had enjoyed good health till two days after a debauch, when he came into the Vienna hospital with symptoms of gastric fever. He then presented symptoms of cerebral congestion, complained of thirst and nausea, and had much abdominal tension. The tongue was loaded; the pulse full, hard, and frequent. Diarrhœa which had been present, had ceased. On the following day, the whole surface of the body, with the exception of the face, was covered by an erythematous eruption, intermixed over the abdomen with purple patches. The respiration was anxious and hurried; the epigastric region tense and painful; the pulse frequent and

* Bull. Gén. de Thérap. t. xxv, p. 5.
† Bull. de l'Acad. Oct. 1843.
‡ Medical Gazette, 1842-3-4.
§ Lancet, vol. ii, 1842-3, p. 65.
|| Clinical Lecture, reported in Provincial Medical and Surgical Journal, Nov. 5, 1842.
** Medical Gazette, July 7, 1843.
†† Il observatore Medico, Feb. 1842.
‡‡ Gazette des Hôpitaux, Oct. 20, 1842.
§§ Provincial Medical and Surgical Journal, June 17, 1843.
|||| Ibid. No.149.
*** New York Journal of Medicine, May 1844, p. 369.
††† Oesterreich. Med. Woch. 1843, No. 16.

full; and the diarrhœa had returned. Infusion of ipecacuanha with Haller's acid elixir was ordered. In the evening there was much febrile exacerbation, with constant jactitation, and the erythema was replaced by a petechial eruption, the patches of which were largest and most numerous over the abdomen, whence they rapidly extended, and assumed a violet colour. The skin was hot, dry, and pungent to the touch. The following day the face was swollen "and ferocious," and the conjunctivæ of a ruby red, from suffused blood. The tongue, gums, and fauces were white as though they had been pencilled with nitrate of silver; the breath was burning, and there was intense thirst, anxious respiration, epigastric and abdominal tension, diarrhœa, pale turbid urine, with much mucous sediment, and the purpura had extended to the knees and neck. On the chest the sebaceous follicles were prominent, and their contents readily oozed out on pressure. The following day these symptoms were present in a still more aggravated form, and there was cough with sanguineous expectoration; extreme oppression, and general insensibility came on, and the patient died in 28 hours from the first appearance of the petechiæ. The mucous and serous membranes were throughout covered with ecchymoses, and the cellular tissue infiltrated with bloody serum, which also was effused into the pleural and pericardial sacs. The lungs and liver were largely infiltrated with blood; the spleen was voluminous but firm, and of a brownish red colour. The intestinal follicles were tumefied. The thick, dark-coloured blood which existed in the vena portæ was, on analysis, found to be deficient in fibrine and salts. [Was this merely a malignant form of fever, and does the condition of the intestinal follicles support this view? Or, was it an example of the petechial smallpox described by Moreton, Sydenham, and Heberden?] A very similar case is described by Mr. Adams,* as probably arising from the variolous contagion to which the patient had been exposed some weeks previously. There was no appearance of vesicles, pustules, or papulæ; the eruption was nowhere elevated above the surface. There were also bloody discharges from the bowels and bloody urine. Five days, from the commencement of the attack, elapsed before death took place. Three cases detailed by Dr. Wotherspoon,† bear also a close analogy to the above. They occurred in the New York hospital in three successive winters, and are described as "a rare form of exanthematous disease." They all occurred in strong healthy men in the prime of life, and in all, the symptoms and pathological appearances were very similar. The prodromi were those of a severe febrile attack, and the succeeding phenomena bore the closest resemblance to those above detailed in Dr. Hummel's case, with the exception that there were ecchymoses observed about the pharynx during life, and numerous minute vesicles on the surface filled with bloody serum, and hemorrhagic discharges from all the outlets of the body. Slight delirium preceded death, which occurred on the fourth or fifth day. The blood had the appearance of a dark cherry red watery fluid, and contained merely a few thin friable black coagula. From the character of the premonitory symptoms, and of the eruption, as well as from the variolous fetor exhaled, and the strong resemblance which the cases bore to the peculiar forms of variola described by the older writers, the author is disposed to refer these cases to variola.

Mr Stainthorpe reports a case of purpura hæmorrhagica occurring in a stout healthy-looking child, æt. 4, and attended with febrile symptoms, in which acetate of lead and opium with wine appear to have been very useful.‡ Mr. Whitwell§ has treated three cases successfully with creosote, [but it may be questioned whether in these instances, occurring in persons almost starving, the cure was not rather attributable to the improved diet.]

Dr. W. Samson Himmelstiern,‖ has described an epidemic of *scorbutus* which prevailed in Russia, in the spring and summer of 1840. Mr. Dalton refers to Dr. Baly's communication (Medical Gazette, February 10) on the antiscorbutic

* Medical Gazette, Jan. 17, 1845.
† New York Journ. of Med. Science, March 1844, p. 203.
‡ Med. Gaz. Oct. 14, 1842.
§ Lancet, Feb. 11, 1843.
‖ Haser's Archiv, v. 4, in Schmidt's Jahrbucher, 1844, No. 8, p. 193.

virtues of the potato, which he confirms by his own observation on board ship, on various occasions.*

*Climacteric disease.* Dr. H. Kennedy† justly observes that nothing has been added to this subject since the original paper of Sir H. Halford, throughout which Dr. Kennedy thinks an erroneous idea prevails, viz. that the affection occurs only in the aged. "At least," he says, "I may state with certainty that an affection which agrees in every respect with climacteric disease is by no means unfrequently met with in individuals between 20 and 30 years of age." His chief object is to direct attention to this point. He alludes to the fact that the various pains which very commonly usher in the attack are in a marked degree periodic. Weakness of the knees is a common symptom, not always connected with or dependent on exercise, but coming on at a particular time, even when the patient is at rest. The disease often sets in with acute symptoms, which are apt to lead astray, particularly when referrible to the head. Loss of sleep is the most constant symptom, and loss of flesh and a marked change in the countenance are also very constant phenomena; but he disagrees with Sir Henry Halford regarding the acceleration of the pulse; having seen cases in which, from first to last, the pulse was not in the least quickened. Partial paralysis (imperfect i. e.) is common. He disagrees with Sir Henry Halford in reference to the renal secretion, which Dr. Kennedy has found diminished, and often depositing the lithates throughout the illness. Men suffer more during the progress of the disease from derangement of the digestive system and brain, and women from symptoms referrible to the lungs or heart. The *average* duration of the complaint is about nine months. In regard to diagnosis, there is danger of confounding many cases at first with local organic disease. Most cases do well, though many are fatal in advanced life. The nervous system is mainly implicated. In opposition to Sir Henry Halford, he thinks the effects of the disease are often entirely shaken off. As it cannot be cut short, too much should not be attempted. Medicines are apt to act *peculiarly*; quinine is one of the most useful. Change of air is not desirable early in the disease. If any indiscretion in diet be committed, it is often followed by an aggravation of symptoms, after 48 rather than 24 hours. Medical treatment is of more avail in the latter half of the illness. [This paper certainly adds many important particulars to Sir Henry Halford's account. The disposition to periodic action, and the calmness of the pulse in some cases, the reporter can confirm from his own observation.] The history of his own case, given by Sir Alexander Crichton, under the denomination of "interrupted circulation," and in which the Bath waters on two occasions were found useful, may perhaps be referred to this head.‡

*Mollities ossium.* Mr. Solly§ has recorded two cases of this somewhat rare disease, both occurring to females, one aged 29 and the other 39. The former died in a state of mental derangement, the membranes and substance of the brain having become implicated in the active disease, which had, for some time, been going on in the bones of the cranium. The immediate cause of death in the latter case was suffocation from contracted thorax. From a comparison of the symptoms during life with the appearances after death, Mr. Solly believes that the disease is of an inflammatory character. The earthy matter of the bones is, he thinks, absorbed and thrown out of the system by the kidneys. The excretion of earthy matter was in one instance so abundant as to have clogged up the calices and pelves of the kidneys, and formed a solid calculus. This excretion of phosphate of lime the author considers not to have been previously established, the chemical constitution of the earthy deposits observed in the urine not having been ascertained. The place of the earthy matter in the bones is supplied by the red grumous matter which abounds in them, and which he believes to be a morbid product, the result of active disease.

* Lancet, vol. i, 1842-3, p. 895.

† Dublin Journal of Medical Science, May 1844; Observations on Climacteric Disease, with Cases, by H. Kennedy, M.D. &c.

‡ Lond. Med. Gazette, Dec. 1, 1843.

§ Two Cases of Mollities Ossium, read before the Med.-Chir. Society, June 18, 1844.

